# Neuro-safety science: an emerging discipline to reveal the neural mechanisms of safety problems

**DOI:** 10.3389/fnins.2023.1190995

**Published:** 2023-08-10

**Authors:** Shu Zhang, Shufen Ye, Yunfang Huang, Xiuzhi Shi

**Affiliations:** ^1^School of Resources and Safety Engineering, Central South University, Changsha, Hunan, China; ^2^Safety & Security Theory Innovation and Promotion Center, Central South University, Changsha, Hunan, China

**Keywords:** safety and security science, neuroscience, neuro-safety science, theory, application

## Abstract

At present, the research of safety science discipline is limited to the level of describing psychology and behaviors, because the cognitive neural mechanisms behind them are unknown. This paper introduces an emerging interdiscipline, namely neuro-safety science, which uses the neuroscientific methods to investigate the neural systems behind safely relevant behaviors. Qualitative methods such as literature review method and theoretical model construction method were adopted for this study. Based on the background of neuro-safety science, the definition of neuro-safety science was defined, its connotation was analyzed, and the research contents from two aspects of theoretical research and practical application research were proposed. Methodology system including research principles, research routes, research procedure and research methods, and the paradigm system of neuro-safety science were put forward. At last, the application research on neuro-safety science was forecasted. This paper opens up a new research perspective for the research of safety science, and provide guidance and reference to develop neuro-safety science.

## 1. Introduction

In human survival, life and production activities, people are the implementers of unsafe behaviors and also the bearers of accidents. Eliminating, controlling or reducing unsafe behaviors is of great significance for safeguarding human life safety. Human behavior is the external manifestation of internal psychological activities (Zhao, 2012), while psychological activities are the manifestation of neural activities (Shi, 2011). Fleischer et al. (2012) pointed out that if we want to perceive the relationship between internal psychology and external behavior, we must rely on potential neural mechanisms. For the various unsafe behaviors caused by various psychological factors, it is a major demand of today’s society to deeply reveal the basic mechanisms of various psychological phenomena and unsafe behaviors at the neural level. In addition, how to quantitatively explain the relationship between psychological phenomena and unsafe behaviors, how to use objective indicators to reflect the influence mechanism of relevant variables are the key problems to be solved in the field of safety science. Thanks to the development of neuroscience, it is possible to deeply reveal the fundamental mechanisms of various psychological phenomena and unsafe behaviors at the neural level, so as to do psychological guidance and behavior control fundamentally.

In recent years, neuroscience has been widely used to study the neural mechanisms behind human behavior, such as organizational behavior (Li et al., 2016), purchasing behavior (Ma et al., 2007), management behavior (Ma and Wang, 2006a,b). It has integrated with relevant disciplines to form a series of interdisciplinary disciplines, such as neurohistology (Becker and Cropanzano, 2010), neuroeconomics (Yu and Zhou, 2007), and neuromarketing (Neurology, 2004). At the same time, the methods and technologies of neuroscience also provide a new direction for the research of safety field. For example, some researchers use neuroscience technology to study the physiological and psychological mechanisms and influencing factors of unsafe behaviors of drivers (Liang and Lin, 2018; Watling and Home, 2022) and construction workers (Liao et al., 2021; Chen et al., 2022). Some have revealed the neural mechanisms of fear emotion infection, cognitive bias, emotional motivation and other influences on decision-making under emergencies (Feng, 2013; Huang, 2014; Shi, 2018); Bian and Jin explored the individual’s neurocognitive process of safety signs and signal words (Jin, 2011; Bian, 2014).

However, there is still a lack of research on the systematic discussion of interdisciplinary integration from the perspective of disciplines. The research of safety science can meet the most basic and fundamental safety needs of human beings, while neuroscience represents the forefront of modern scientific research. These two disciplines will be integrated. Therefore, the field of safety science can be regarded as a new extension area of neuroscience, and it is proposed that neuro-safety science will open up a new perspective to study safety science issues.

## 2. Research background of neuro-safety science

### 2.1. The needs of social development

In daily safety management, measuring and judging the match between individual psychological characteristics and safety work has become a requirement to be met (Yang and Zhang, 2019; Zhou, 2021). Based on objective measurement of individual personality and psychological characteristics by the methods and techniques of neuroscience, it can be achieved: firstly, to develop physiological and psychological measurement indicators for job requirements in specific positions such as high-risk and high-pressure, for selecting job practitioners. Secondly, to conduct job matching measurement during job recruitment, and hire individuals who are more suitable for the job. Thirdly, in daily work, regularly to carry out job adaptability measurements to determine the compliance between employees and their positions in real-time. Finally, when conducting safety activities such as education and training, analyzing the EEG data obtained from individual neural measurements can objectively and concretely evaluate the effectiveness of safety training. In summary, it can be seen that social development requires the research results of neuro-safety science.

### 2.2. The needs of discipline development

It is important contents of safety science to clarify the internal mechanisms of safety-related psychology and behavior to guide unsafe psychology and eliminate unsafe behaviors (Sui, 2000). Safety psychology and safety behavior are two branches of safety science, and their comparison with neuro-safety science is shown in Table 1. As far as human society is concerned, security needs exist in the whole field of human activities (Wu and Wang, 2018). Horizontally, neuro-safety science extends its research subject to all members of society, and its research scope extends to the whole space of human activities. In the longitudinal direction, the research of neuro-safety science goes deep into the neural level, which can obtain objective quantitative data, explain and verify the relevant theories of safety psychology and safety behavior from a new perspective, reveal the internal laws and neural mechanisms of human safety-related psychology and behavior. Therefore, from the perspective of the in-depth research needs of safety psychology and behavior mechanisms, the construction of neuro-safety science is a historical necessity for expanding and deepening safety science.

**Table 1:** Branch disciplines of safety science that research psychology and behavior.

Disciplines	Safety psychology	Safety behavior	Neuro-safety science
Time	In the early 20th century	The 1980s	2023
Origin	Occupational health psychology	Behavior science, behavior safety management	Neuroscience, safety science
Research contents	Explore the psychological phenomena related to unsafe behavior in the process of labor and production	Study the problems related to people’s psychological and physiological behaviors and safety, reveal the laws of people’s behaviors in the work and production environment	Research on the brain neural mechanisms of psychological phenomena and behavioral activities related to safety in human activities
Research methods	Investigative method, psychological measurement, experimental methods, etc.	Observation, inquiry, physiological measurement, etc.	Psycho-physiological measurement, Brain Imaging and EEG Measurement Technology, etc.
Theories	Thurley model, cognitive failure theory, etc.	Goal theory, S-O-R-A behavior model, two-factor theory, etc.	Risk perception two-stage model, risk decision-making three-phase conceptual model, etc.

Besides, now neuro-safety science is an interdiscipline that extends neuroscience to the field of safety science to study unsafe psychology and unsafe behavior. Therefore, from the perspective of multidisciplinary and multi-level development needs of neuroscience (Zhang H. L. et al., 2021; Zhang S. et al., 2021), the construction of neuro-safety science is an important opportunity for neuroscience to broaden its research scope and application fields.

## 3. Definition and research contents of neuro-safety science

### 3.1. Definition of neuro-safety science

#### 3.1.1. Definition

Neuro-safety science is a new interdisciplinary subject involving neuroscience, safety science, psychology and behavioral science. Through the technical methods of neuroscience, the brain neural mechanisms of safety-related psychological phenomena and behavioral activities in human activities are studied, and the high-level psychological micro-process is revealed, which focuses on the cognitive processes such as attention, feeling and memory of danger, and its relationship with behavioral decision-making and the law of behavioral development, so as to describe, explain, predict, guide and control human psychology (Zhang S. et al., 2021) and behavior and finally promote human psychological and behavioral safety. In order to better understand the definition of neuro-safety science, the connotation of neuro-safety science are elaborated from three aspects: discipline orientation, research basis, and research subjects.

#### 3.1.2. Connotation of definition

##### 3.1.2.1. Discipline orientation

Neuro-safety science is a branch discipline of safety science. It is an interdiscipline integration of safety science and neuroscience. Safety is the research aim of safety science, which refers to the psychological and behavioral safety of individuals, including both physical protection from external hazards and mental health not being threatened (Wu et al., 2018). Nerve is the research unit of neuroscience and is the source of human psychological phenomena and behavioral activities. Neuro-safety science is based on the principles and methods of safety science, with neuroscience techniques as the main method to study the neural mechanisms related to safety psychology and behavior.

##### 3.1.2.2. Research basis

**Figure 1:**
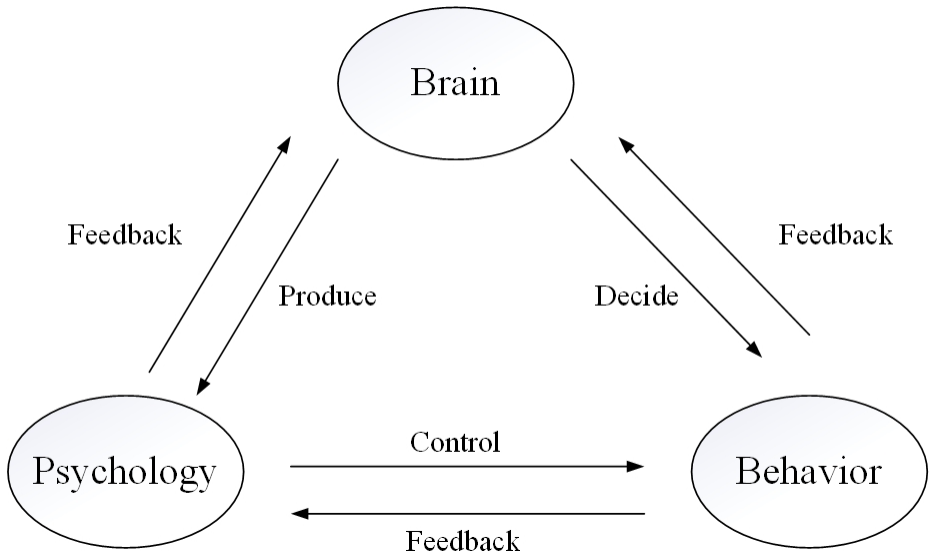
The relationships of brain, psychology, and behavior.

Human psychological phenomena and behavioral activities have their corresponding neural mechanisms at the brain nerve level. The relationship between brain, psychology and behavior is shown in Figure 1. In the current study, the neural mechanism is mostly characterized by EEG component, temporal and spatial distribution and connection of brain area, activation state and level of brain area, etc. For example, Teng et al. found that aggressive behavior was related to early auditory P50 wave, error-related negative wave and late P300 wave, and the corresponding activation of brain areas included prefrontal lobe, anterior cingulate gyrus and amygdala (Teng et al., 2013). As the supreme headquarters of psychological and behavioral activities, the brain controls almost every behavior of humans. On the one hand, different behaviors have corresponding main brain regions in the brain. For example, Camerer et al. (2004) found that risk aversion behavior caused by fear was largely related to the amygdala. On the other hand, there is corresponding brain activity in the brain before and after behavior execution. For example, before individuals make risky decisions, the nucleus accumbens is activated, When individuals tend to conservative behavior, the hypothalamus is more active (Kuhnen and Knutson, 2005). Therefore, human psychological phenomena and behavioral activities have corresponding neural mechanisms at the brain level. Neural mechanisms are the focal point of neuro-safety science research.

##### 3.1.2.3. Research subjects

Establishing the research subjects of a discipline is the most fundamental and essential task for the development of that discipline (Wang and Wu, 2019). The research subject of neuro-safety science is human beings, specifically individuals, groups, and organizations. At the individual level, the focus is on studying the relationship between an individual’s psychology and behavior, as well as the corresponding neural mechanisms. At the group level, the main research is on the interaction mechanisms between individuals and exploring the micro processes of psychological states and their mutual influence among people. At the organizational level, the research examines the interrelationships among groups within organizations and the impact of organizational factors on group psychology and behavior. Through these three levels, a comprehensive exploration of the psychological and behavioral neural mechanisms related to safety can be conducted.

### 3.2. Research contents of neuro-safety science

The most important aspect of creating a new discipline is the establishment of its foundational theories (Wu, 2021). This is also true for the establishment of neuro-safety science. It is essential to conduct basic theoretical research of the discipline. Thus, in the theoretical research, the basic issues, the disciplinary system and the methodology system of neuro-safety science must be studied. It can further guide the construction of the discipline system, and will comprehensively examine the practical application of neuro-safety science from a theoretical perspective.

According to the definition, research basis and research aim of neuro-safety science, it is necessary to conduct practical application research from both the individual micro level and the organizational macro level. At the micro level, this study investigates the relationship between individual physiology, psychology, and behavior involving safety, as well as their internal neural mechanisms. At the macro level, it includes studying the behavioral characteristics and internal neural mechanisms of groups and organizations, and finally applying neuro-safety science to various fields of safety to achieve the goal.

In summary, the research contents of neuro-safety science include two aspects: basic theoretical research and practical application research, as shown in Table 2.

**Table 2:** Research contents of neuro-safety science.

Research classification	Research contents	
Basic theoretical research	Study the basic issues of neuro-safety science. It includes the definition of discipline, its connotation, attributes and characteristics, research subjects, research content and research aim, etc.	
	Study the disciplinary system of neuro-safety science. It includes discipline basis, connection with relevant disciplines, discipline orientation, main discipline branches, etc.	
	Study the methodology system of neuro-safety science. It includes an overview of the methodology, the research principles, routes, procedure and methods of neuro-safety science.	
Practical application research	The micro level	Study the relationship and characteristics of individual physiology, psychology and neural activities. Explain the internal neural mechanisms of the interaction between individual physiology and psychology.
		Study the relationship between individual psychology and behavior and its neural mechanisms. Analyze the behavioral manifestations corresponding to different psychological states of individuals, and reveal the relationship between individual high-level psychological micro-process and behavior decision-making.
		Study the impact of individual subjective internal factors and objective external environmental factors on individuals’ unsafe psychological state and behavioral activities, and reveal the internal neural mechanisms of their influence. Individual psychological factors such as risk preference, personality psychology, emotional state, etc., external factors such as social environment, natural environment, etc.
	The macro level	Study the psychology and behaviors characteristics of different individuals and groups and the interaction mechanisms of the group. It aims to achieve the guidance of psychological state, and the prediction, guidance and control of possible behavior trends.
		Study the psychological and behavioral characteristics of different organizations, including the impact mechanisms of organizational culture, systems, policies, etc. on individual psychology and behavior within the organization.
		Study on the practice and application of neuro-safety science in various fields of safety, such as the application of emergency management in public safety to guide individual emergency safety behavior, the application of worker safety management in industrial safety to improve workers’ risk perception ability, etc.

## 4. Methodology of neuro-safety science

### 4.1. Definition of the methodology neuro-safety science

Methodology, summarized the experience of human creation and use of various methods from a philosophical perspective, is a doctrine about research methods. It affects and restricts the selection and application of methods, and provides inspiration and guidance for methodological research (Wu, 2011). Any discipline will generate a set of distinctive research principles, routes, procedure, and theories based on its own characteristics, and form a methodological system to guide the development of discipline research (Cao, 2014). Therefore, the theoretical research of any discipline cannot be separated from the study of its methodology.

**Figure 2:**
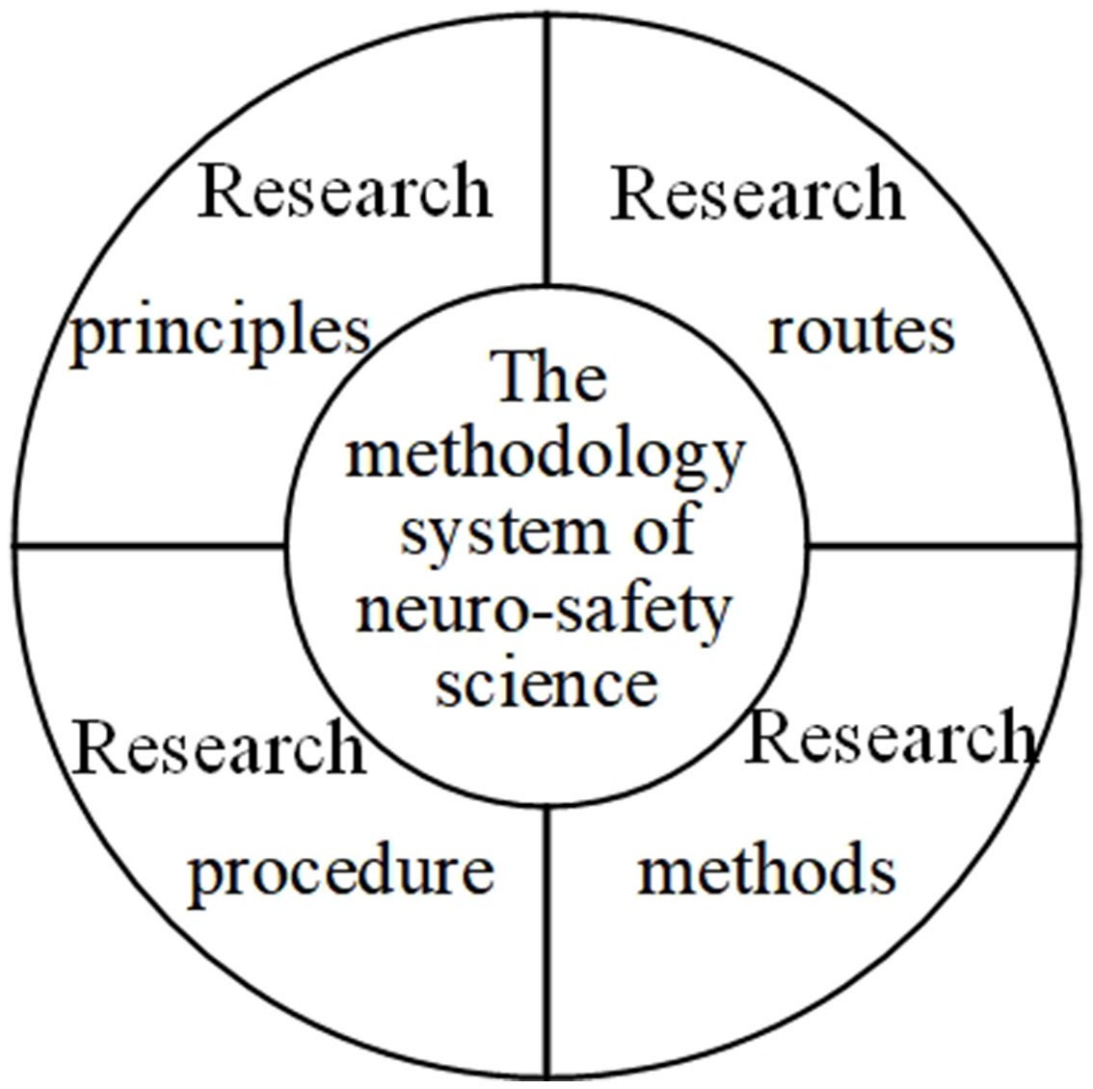
“Four in one” methodology system of neuro-safety science.

The methodology of neuro-safety science is a general theoretical orientation used to guide the research of neuro-safety science. It is based on philosophy, safety science methodology and core principles (Zhang et al., 2022) and other theories. It includes the general principles and procedures, routes and methods to be followed by the discipline research from a theoretical and philosophical perspective, as shown in Figure 2. The methodology of neuro-safety science helps researchers understand the process of neuro-safety science research to the greatest extent.

### 4.2. Methodology system of neuro-safety science

The research principles, research routes, research methods and research procedure of neuro-safety science do not exist independently. They interact and promote each other, and jointly form the methodology system of neuro-safety science, which play a theoretical guiding role in the development of discipline research.

#### 4.2.1. Research principles

According to the discipline characteristics and research contents of neuro-safety science and the core principles of materialist dialectics, neuro-safety science research should follow four basic principles.

(1) Principle of individual research and group research.

Individuals and groups have mutual influence and interaction in psychological phenomena and behavioral patterns (Wang and Yu, 2016). The practical application research of the research content is mainly divided into two levels: the micro level mainly focuses on individuals, while the macro level focuses on groups and organizations. Therefore, it is essential to adhere to the principle of individual research and group research when conducting research on neuro-safety science.

(2) Principle of longitudinal research and cross-sectional research.

When conducting individual research, different individuals have different psychological and behavioral activities under the same conditions, and the same individual also has different psychological and behavioral activities under the same conditions at different ages. Therefore, it is important to adhere to the principle of longitudinal research and cross-sectional research (Salthouse, 2019).

(3) Principle of qualitative research and quantitative research.

According to the disciplinary definition of neuro-safety science, it mainly studies the brain neural mechanisms of psychological phenomena and behavioral activities related to safety in human activities. This requires a description of their properties, characteristics, manifestations, etc., as well as objective quantitative data obtained through experimental research. Therefore, the principle of qualitative research and quantitative research is indispensable.

(4) Principle of attribution research and prediction research.

Description, interpretation, prediction, guidance and control are a set of objective processes of neuro-safety science. To achieve these, on the one side, it is necessary to explain and infer the psychological motivation and behavioral processes of the research subjects, as well as its influencing factors, and to search for its neural mechanisms; On the other, it is vital to predict unknown possible psychological and behavioral manifestations based on research results for further guidance (Zhang et al., 2020). Therefore, the principle of attribution research and predictive research must be adhered to.

#### 4.2.2. Research routes

**Figure 3:**
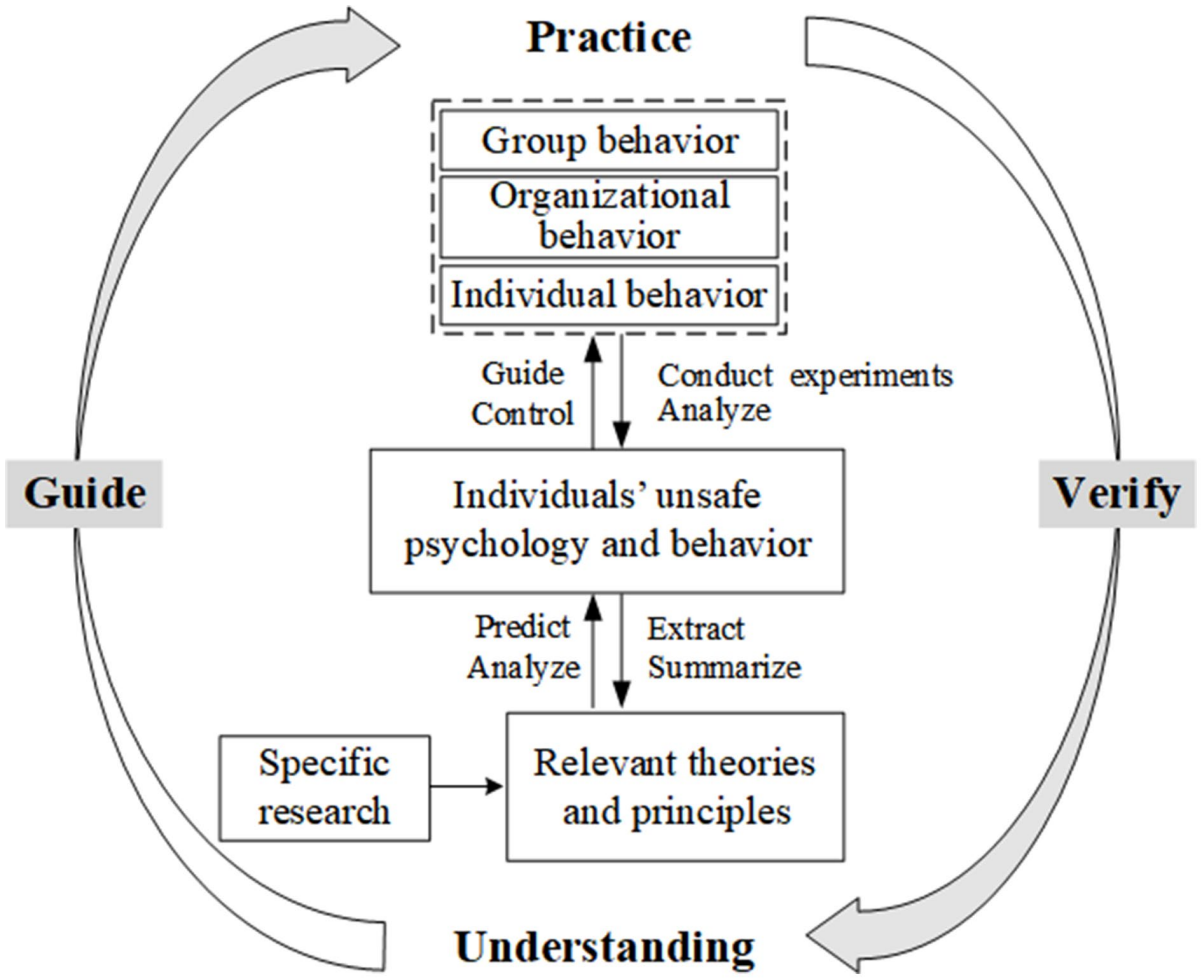
Research routes of neuro-safety science.

It is an iterative process from practice to understanding to practice. “From practice to practice” is the content of dialectical materialism epistemology and the philosophical approach of scientific research (Zhou, 2016). The research of any discipline should follow the philosophical approach of scientific research. Therefore, research routes of neuro-safety science discipline research are correspondingly proposed (see Figure 3).

(1) Research route of “from understanding to practice.”

Relevant theories and principles of neuro-safety science are summarized from specific research, which include the fundamental theories of safety psychology and behavior, neural mechanisms, and so on. Under the guidance of theories, potential unsafe psychological and behavioral tendencies of individuals under different conditions are predicted. And then practical research on specific problems related to individuals, groups, organizations, etc., can be smoothly carried out. Therefore, theoretical understanding research is a necessary basic research that can provide important guidance for practical research.

(2) Research route of “from practice to understanding.”

Experimental research on the psychology and behavior of individuals, groups, and organizations is conducted, and the unsafe psychology and behavior of individuals are analyzed. And the research results can present two situations. When the research results support the original theory, empirical evidence is added to the theory. When the research results contradict the original theory, they may overturn or question the original theory, and there may be new discoveries. This process plays a validation role in theoretical understanding research.

#### 4.2.3. Research methods

Neuro-safety science is an interdisciplinary discipline formed by introducing the technology of neuroscience into the field of safety science. Brain imaging and EEG measurement technology are currently the most advanced and accurate research methods in neuroscience research. The emergence and development of these technologies have made it possible to open the “black box” of the brain and directly observe the activities inside the brain without damage (Liu and Tian, 2012). This provides scientific and objective experiment measurement for neuro-safety science to explore the brain neural mechanisms behind psychological behavior.

Meanwhile, the development of neuro-safety science needs the help of the methods, technologies and experiences of other disciplines, as shown in Table 3.

**Table 3:** Research methods and application of neuro-safety science.

Methods	Application in neuro-safety science
Experiment measurement	Measure the composition and changes of human EEG, the spatiotemporal distribution and connectivity of brain regions, the activation status and level of brain regions, etc. with EEG measurement technology and brain imaging measurement technology.
Hypothesis verification	Propose research hypotheses based on the research aims, then design neuro-safety experiments, and determine whether the hypotheses have been verified based on the experimental results.
Observation – interview	Study human behavior, activity patterns, emotional states, reaction abilities, personality traits, risk preferences, etc.
Comparative analysis	Compare the experimental results of different subjects (with different variables) or the same subject at different stages (under different initial conditions) using EEG components, activation status and level of brain regions as indicators.
Induction deduction	Summarize neuro-safety science theories such as discipline principles, basic rules, discipline models, discipline concepts, and discipline application theories. Predict individual and group psychological and behavioral trends based on disciplinary theories.
Prediction analysis	Predict and grasp the possible behavioral trends of different individuals and groups in different situations by analyzing phenomena and patterns, in order to do psychological counseling and behavioral guidance better.

#### 4.2.4. Research procedure

**Figure 4:**
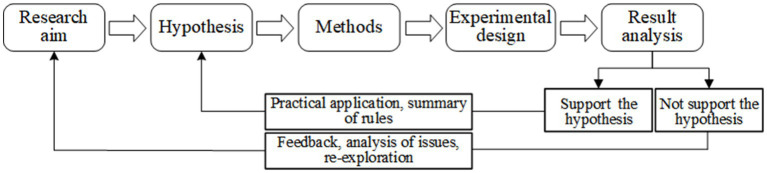
Research procedure of neuro-safety science.

Neuro-safety science research involves a series of orderly steps. The general research procedure of neuro-safety science is shown in Figure 4.

Step1: Determine research aim. Researchers should clarify research aims based on research background and research questions firstly. For example, studying the influencing factors of safety risk perception is an aim to be studied.

Step2: Put forward hypothesis. Researchers need to conduct comprehensive analysis in combination with the theoretical principles of neuro-safety science and relevant practical experience, construct the research hypothesis, and then start scientific research on neuro-safety science. For example, it is assumed that emotions are one of the influencing factors of safety risk perception.

Step3: Select methods. Researchers should select appropriate research methods to verify the research hypothesis. For example, in order to better and more objectively study the impact of emotions on risk perception, experimental methods were chosen instead of questionnaire surveys.

Step4: Design experiment. The experimental research is the most basic and central research method of neuro-safety science. And the experimental design includes paradigm selection, scheme design, experimental implementation and data analysis.

Step5: Analyze results. Researchers analyze the research results, summarize and reflect on the relevant practical experience, and provide new ideas and opportunities for new research in neuro-safety science. That is, if the hypothesis is not supported, the research results are directly fed back to the research aim stage, so that researchers need to re-explore research aims. If the hypothesis is supported, researchers can summarize the rules from the research conclusions, and ultimately integrate them into the theoretical principles of the discipline.

### 4.3. Research paradigm of neuro-safety science

**Figure 5:**
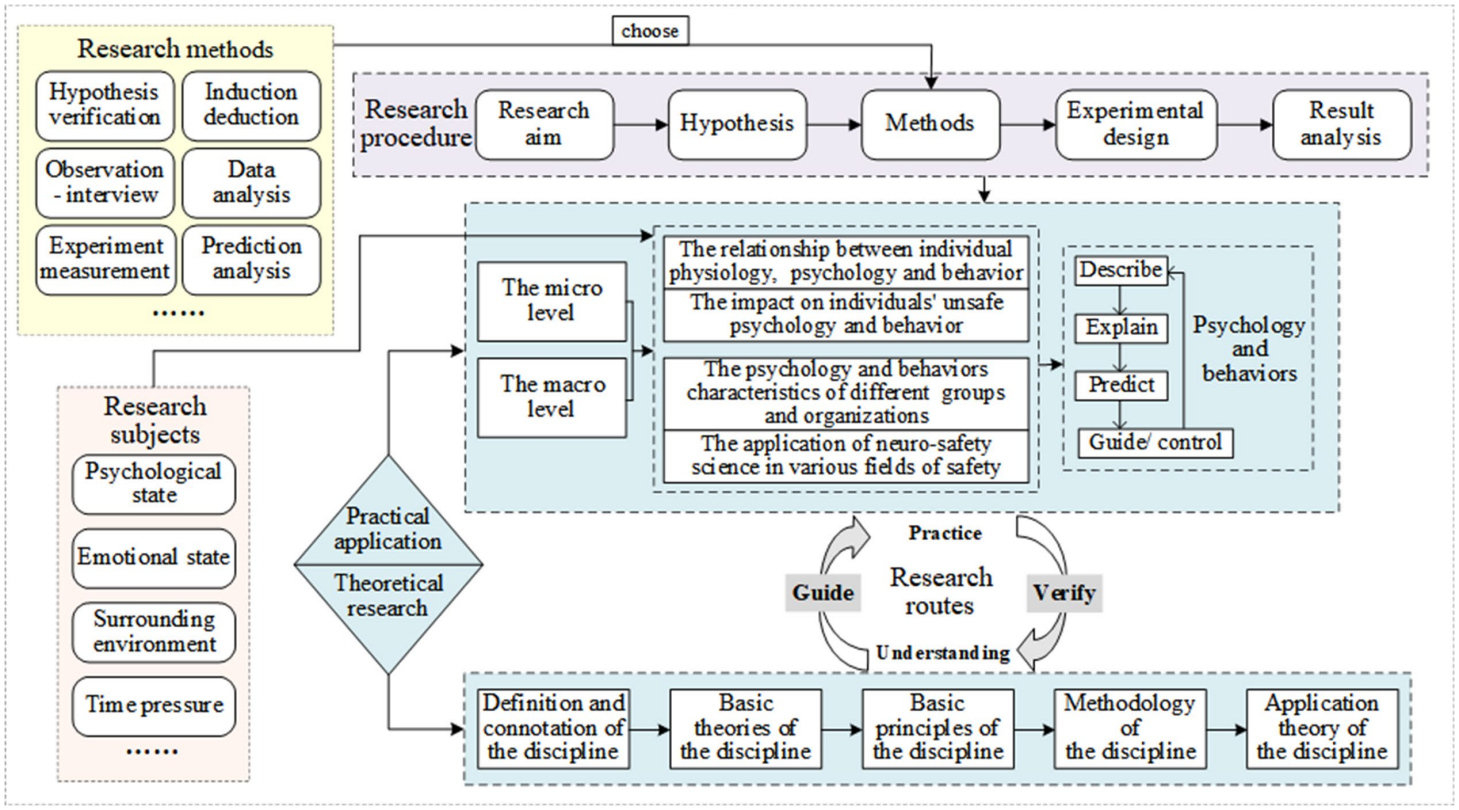
Research paradigm of neuro-safety science.

Combining the methodology system and research contents of neuro-safety science, the research paradigm system of neuro-safety science was constructed (see Figure 5).

1. The research subject of neuro-safety science is an important foundation for conducting research. The internal factors such as personality psychology, emotional state, and psychological state of them, as well as external factors such as surrounding environment and time pressure, will affect the research results. In practical applications, strategies should be adjusted appropriately according to the different research subjects.
2. The research contents of neuro-safety science are divided into theoretical research and practical application research. The research of discipline theory starts from the most basic definition and connotation of discipline, and proceeds along the route of “basic theory - basic theory of discipline construction - discipline methodology - discipline application theory” (Cao, 2014). The practical application research of discipline starts from the specific research problem type and follow the route of “experiment - application - summary.” The research including “individual micro level and the organizational macro level” and should be carried out along the research routes with appropriate research methods. Finally, the application objective is to “describe, explain, predict, guide and control human psychology and behaviors.”
3. Theoretical research guides practical application research, while practical application research validates theoretical research. The ultimate purpose of their synergy is to improve the discipline system of neuro-safety science, promote the scientific development of it, fill the gaps in the research of human psychology and behavior related to safety in the field of safety science, and promote the safety of human psychology and behavior.

It is worth emphasizing that this research paradigm is the basic framework for the research of neuro-safety science, which regards the research of neuro-safety science as a complete system of interaction. It plays a driving role in the construction of the discipline system of neuro-safety science.

## 5. Application research of neuro-safety science

Neuro-safety science emphasizes that under the guidance of basic theories, researchers use the latest technology and instruments of neuroscience, and take experimental research as the main means to focus on the brain and neural mechanisms of safety-related psychological phenomena and behavioral activities in the whole range of human life, production and survival activities. Thus, neuro-safety science has extensive range of application.

Application in ergonomics. The theories and technologies of neuro-safety science could applied to design the human-machine systems, and pay attention to the interaction between human and environment, and between human and machine. Many existing studies have used ERP and fMRI to discover the neural mechanisms underlying individuals’ cognitive processes towards various safety signs (Qin and Han, 2009; Ma et al., 2010; Tang, 2010; Jin, 2011; Bian, 2014; Lu and Zhang, 2020; Wu et al., 2022). There are many studies to explore the effectiveness of safety signs with ERP from the perspective of safety sign design (Tang, 2010; Bian, 2014; Han, 2019; Wang, 2019; Zhao et al., 2022). However, there are still many production environment factors and machine design that have not been studied, such as equipment layout, lighting, ventilation and essential safety of machines, etc., which needs further improvement.

Application in management. Personnel management is the core of safety management. The theories and technologies of neuro-safety science are conductive to explore the impact of various cognitive factors on behavior among workers, including risk appetite (An, 2015), fatigue (Xing et al., 2020), cognitive load (Liao et al., 2021), incentive (Qin, 2013), emotional state and attention (Wang et al., 2017), etc. There have been a large number of studies on human risk cognitive with neuro-safety science, but a complete research system has not been formed. The brain neural mechanisms of human risk cognition and behavioral decision-making, as well as the influencing mechanisms of various internal and external factors, need to be further improved.

Application in risk evaluation. Safety evaluation includes two parts: identification of the risk factor and evaluation of risk degree. Currently, Ma et al. (2014) applied the research methods of neuroscience to the study of safety risk cognition and proposed a two-stage model for safety risk cognition, which involves later evaluation of risk level. Qin and Han (2009), Jin (2011), and Fu (2016) have demonstrated this model. Wetton et al. (2010) proposed a three-stage model for driver hazard identification, including hazard evaluation. However, the underlying neural mechanisms of risk evaluation, as well as the influencing mechanisms of influencing factors, are currently unclear.

Application in safety education. At present, some scholars have constructed a pedestrian (Dommes and Cavallo, 2012) and driver (Gianclaudio et al., 2014) hazard perception training system. But, safety education is mainly divided into three categories: professional safety education, safety training and education, public safety education. With the help of neurosafety theory and technology, the neural mechanisms of these safety education and the impact neural mechanisms of influencing factors need to be studied.

Application in safety prediction. Predicting human psychology and behavior is an important goal of neuro-safety science. The general mechanisms of understanding the development and change of human safety psychology and behavior should be summarized by using neuro-safety science and technology. Zhang et al. (2023a,b) studied the influence neural mechanisms of emotion on risk perception and unsafe behavior decision-making, and established a model to provide new ideas for predicting unsafe behavior in humans, in order to prevent accidents caused by emotions. But there are many factors that affect unsafe behavior, and it needs to be explored step by step in order to better predict it.

In addition to the above, there are many other aspects of application research of neuro-safety science. All application researches related to human safety belongs to the range of neuro-safety science. Therefore, the development of neuro-safety science needs more researchers to carry out more application research.

## 6. Conclusion

Neuro-safety science is an inevitable branch discipline of safety science and neuroscience. This paper put forward the definition and connotation of neuro-safety science, clarified the main research contents of it, formed a methodology system of neuro-safety science, and also constructed a research paradigm combining theoretical research and application practice, which laid a solid theoretical foundation for the development of neuro-safety science. Finally, we summarized the research scope and contents of the application practice of neuro-safety science. This paper established the disciplinary framework of neuro-safety science, and pointed out the direction for the development of it.

Therefore, this paper only constructs the general framework of neuro-safety science. The specific connotation of some theories needs to be further studied, and the relevant theories should be tested in the practice of safety work, so that the theories can really guide the development of discipline practice. In addition, this paper focuses on qualitative research, and the development of neuro-safety science needs to increase quantitative research in the future.

## Data availability statement

The original contributions presented in the study are included in the article/supplementary material, further inquiries can be directed to the corresponding author.

## Author contributions

SZ, SY, and YH contributed to conception and design of the study. XS organized the database. SZ wrote the first draft of the manuscript. SY and YH wrote sections of the manuscript. All authors contributed to manuscript revision, read, and approved the submitted version.

## Funding

This research was funded by the Natural Science Foundation of Hunan Province, grant number 2021JJ40801.

## Conflict of interest

The authors declare that the research was conducted in the absence of any commercial or financial relationships that could be construed as a potential conflict of interest.

## Publisher’s note

All claims expressed in this article are solely those of the authors and do not necessarily represent those of their affiliated organizations, or those of the publisher, the editors and the reviewers. Any product that may be evaluated in this article, or claim that may be made by its manufacturer, is not guaranteed or endorsed by the publisher.

## References

[ref1] An Q. W. (2015) Research on the impact of risk preference on unsafe behavior of miners. Master's thesis. Xi'an, Shaanxi: Xi'an University of Science and Technology

[ref2] Becker W. J. Cropanzano R. (2010). Organizational neuroscience: the promise and prospects of an emerging discipline. J. Organ. Behav. 31, 1055–1059. doi: 10.1002/job.668

[ref3] Bian J. (2014) Research on perception mechanism of safety signs in safety management: A neuro-industrial engineering perspective. Master's thesis. Hangzhou (Zhejiang): Zhejiang University

[ref4] Camerer C. Loewenstein G. Prelec D. (2004). Neuroeconomics: how neuroscience can inform economics. J. Econ. Lit. 43, 9–64. doi: 10.1257/0022051053737843

[ref5] Cao Y. Y. (2014) Subject attributes and methodology and application of comparative safety studies. Master's thesis. Changsha (Hunan): Central South University

[ref9002] Chen J. J. Xu Q. W. Fang D. P. Liao P.-C. (2022). Perceptual decision-making ‘in the wild’: How risk propensity and injury exposure experience influence the neural signatures of occupational hazard recognition. International journal of psychophysiology: official journal of the International Organization of Psychophysiology. 177, 92–102. doi: 10.1016/j.ijpsycho.2022.04.012 35569600

[ref7] Dommes A. Cavallo V. (2012). Can simulator-based training improve street-crossing safety for elderly pedestrians? Trans. Res. Part F Psychol. Behav. 15, 206–218. doi: 10.1016/j.trf.2011.12.004

[ref8] Feng Y. D. (2013) Research on information frame effects under paroxysmal events based on brain potential analysis. Master's thesis. Hangzhou (Zhejiang): Zhejiang University

[ref9] Fleischer F. Christensen A. Caggiano V. Thier P. Giese M. A. (2012). Neural theory for the perception of causal actions. Psychol. Res. 76, 476–493. doi: 10.1007/s00426-012-0437-9 22535418

[ref10] Fu H. J. (2016) Research on risk perception based on EEG signal analysis. Master's thesis. Hangzhou (Zhejiang): Zhejiang University

[ref11] Gianclaudio C. Nathan T. Mike M. Martin K. Lutz J. (2014). The drive-wise project: driving simulator training increases real driving performance in healthy older drivers. Front. Aging Neurosci. 6:85. doi: 10.3389/fnagi.2014.00085 24860497 PMC4026721

[ref13] Han Y. Q. (2019) Research on optimization of plant safety signs based on neuroindustrial engineering. Master's thesis. Qinhuangdao (Hebei): Yanshan University.

[ref14] Huang Y. J. (2014) The physiological mechanism of fear transmission after an unconventional emergency occurs. Doctoral thesis. Hangzhou (Zhejiang): Zhejiang University.

[ref16] Jin J. (2011) The risk information processing for warning signal words: An experimental study. Master's thesis. Hangzhou (Zhejiang): Zhejiang University

[ref17] Kuhnen C. M. Knutson B. (2005). The neural basis of financial risk taking. Neuron 47, 763–770. doi: 10.1016/j.neuron.2005.08.008 16129404

[ref9001] Liang B. Lin Y. Z. (2018). Using physiological and behavioral measurements in a picture-based road hazard perception experiment to classify risky and safe drivers. Transportation Research Part F Traffic Psychology \u0026amp; Behaviour. 58, 93–105. doi: 10.1016/j.trf.2018.05.024

[ref20] Liao P.-C. Sun X. L. Zhang D. (2021). A multimodal study to measure the cognitive demands of hazard recognition in construction workplaces. Saf. Sci. 133:105010. doi: 10.1016/j.ssci.2020.105010

[ref19] Li H. Ma Q. G. Dong X. (2016). Neurohistology: conceptual analysis, theoretical development and research prospects. J. Manage. World. 275, 164–173. doi: 10.19744/j.cnki.11-1235/f.2016.08.014

[ref21] Liu X. Tian Y. Q. (2012). Ethical issues in brain imaging technology. Stud. Ethics. 58, 104–108. doi: 10.15995/j.cnki.llxyj.2012.02.027

[ref23] Lu G. Y. Zhang T. (2020). An EEG study on semantic cognition and emotional evaluation for traffic signs. China Safety Sci J. 31, 184–190. doi: 10.16265/j.cnki.issn1003-3033.2021.03.026

[ref24] Ma Q. G. Fu H. J. Xu T. Pei G. X. Chen X. J. (2014). The neural process of perception and evaluation for environmental hazards: evidence from event-related potentials. Neuroreport 25, 607–611. doi: 10.1097/WNR.0000000000000147, PMID: 24686132

[ref25] Ma Q. G. Jin J. Wang L. (2010). The neural process of hazard perception and evaluation for warning signal words: evidence from event-related potentials. Neurosci. Lett. 483, 206–210. doi: 10.1016/j.neulet.2010.08.009, PMID: 20705118

[ref26] Ma Q. G. Shu L. C. Wang X. Y. (2007). Innovative marketing thinking Neuromarketing finds "purchase button". Enterprise Management. 308, 10–13. doi: 10.3969/j.issn.1003-2320.2007.04.002

[ref27] Ma Q. G. Wang X. Y. (2006a). Cognitive neuroscience, neuroeconomics and neuromanagement. J. Manage. World. 10, 139–149. doi: 10.19744/j.cnki.11-1235/f.2006.10.016

[ref28] Ma Q. G. Wang X. Y. (2006b). From neuroeconomics and neuromarketing to neuromanagement. J. Indust. Engin. Engin. Manage. 20, 129–132. doi: 10.3969/j.issn.1004-6062.2006.03.029

[ref29] Neurology T. L. (2004). Neuromarketing: beyond branding. Lancet Neurol. 3:71. doi: 10.1016/S1474-4422(03)00643-4, PMID: 14746993

[ref31] Qin J. G. Han S. H. (2009). Neurocognitive mechanisms underlying identification of environment risks. Neuropsychologia 47, 397–405. doi: 10.1016/j.neuropsychologia.2008.09.010, PMID: 18845172

[ref30] Qin S. Z. (2013). Research on incentive of active individual safety production management based on ERPs experiment. master's thesis. Harbin (Heilongjiang): Harbin Engineering University.

[ref32] Salthouse T. A. (2019). Trajectories of Normal cognitive aging. Psychol. Aging 34, 17–24. doi: 10.1037/pag0000288, PMID: 30211596 PMC6367038

[ref34] Shi R. (2018) Research on the influence of emotional motivation on public behavior decision making in group public emergencies. Doctoral thesis. Qinhuangdao (Hebei): Yanshan University.

[ref33] Shi S. L. (2011). Psychological factors affecting EEG. Guide China Med. 9, 195–196. doi: 10.15912/j.cnki.gocm.2011.30.270

[ref35] Sui P. C. (2000). Safety science and sustainable social development. China Safety Sci. J. 2, 10–13. doi: 10.16265/j.cnki.issn1003-3033.2000.02.002

[ref36] Tang X. W. (2010). Research on safety signs identification via neuroengineering. Master's thesis. Hangzhou (Zhejiang): Zhejiang University.

[ref38] Teng Z. J. Liu Y. L. Pan Y. G. Yao D. Z. (2013). The brain mechanism of aggression: review on ERP and fMRI study. Adv. Psychol. 3, 313–320. doi: 10.12677/ap.2013.36047

[ref42] Wang B. Wu C. (2019). Study on some basic lssues of Safety & Security-related Intelligence Science under the guidance of the big Safety & Security Concept. J. Intelligence 38, 7–14. doi: 10.3969/j.issn.1002-1965.2019.03.002

[ref41] Wang D. Chen J. Y. Zhao D. Dai F. Zheng C. J. Wu X. B. (2017). Monitoring workers' attention and vigilance in construction activities through a wireless and wearable electroencephalography system. Autom. Constr. 82, 122–137. doi: 10.1016/j.autcon.2017.02.001

[ref43] Wang Q. Yu G. L. (2016). The relationship between group identification and individual mental health: moderating variables and mechanisms. Adv. Psychol. Sci. 24, 1300–1308. doi: 10.3724/SP.J.1042.2016.01300

[ref40] Wang Y. F. (2019) Research on safety risk perception of warning signs and their attributes. Master's thesis. Wuhan (Hubei): China University of Geosciences.

[ref9003] Watling C. N. Home P. (2022). Hazard perception performance and visual scanning behaviours: The effect of sleepiness. Transportation Research Part F: Traffic Psychology and Behaviour. 90, 243–251. doi: 10.1016/j.trf.2022.08.020

[ref44] Wetton M. A. Horswill M. S. Hatherly C. Wood J. M. Pachana N. A. (2010). The development and validation of two complementary measures of drivers' hazard perception ability. Accid. Anal. Prev. 42, 1232–1239. doi: 10.1016/j.aap.2010.01.017, PMID: 20441837

[ref45] Wu C. (2011). Safety science methodology. Beijing: China Labor and Social Security Press.

[ref46] Wu C. (2021). Research on basic theory of safety complexity science: laying a foundation for new highland of safety science. China Safety Science Journal. 31, 7–17. doi: 10.16265/j.cnki.issn1003-3033.2021.05.002

[ref48] Wu C. Wang B. (2018). Research review on the trends and theoretical progress of safety science in recent years. J. Saf. Environ. 18, 588–594. doi: 10.13637/j.issn.1009-6094.2018.02.033

[ref49] Wu C. Yang M. Wang B. (2018). Safety definition, intension, extension and inferences from scientific view. J. Zhengzhou Univ. (Engineer. Sci.) 39, 1–4. doi: 10.13705/j.issn.1671-6833.2018.03.002

[ref47] Wu J. C. Du X. X. Tong M. Guo Q. Shao J. K. Chabebe A.. (2022). Neural mechanisms behind semantic congruity of construction safety signs: An EEG investigation on construction workers. Hum. Fact. Ergon. Manufact. Serv. Indust. 33, 229–245. doi: 10.1002/hfm.20979

[ref50] Xing X. J. Zhong B. T. Luo H. B. Rose T. Li J. Antwi-Afari M. F. (2020). Effects of physical fatigue on the induction of mental fatigue of construction workers: a pilot study based on a neurophysiological approach. Autom. Constr. 120:103381. doi: 10.1016/j.autcon.2020.103381

[ref51] Yang G. S. Zhang M. Y. (2019). Research on safety configuration of construction posts based on perspective of people-job fit. J. Engineer. Manage. 33, 138–142. doi: 10.13991/j.cnki.jem.2019.04.026

[ref53] Yu R. J. Zhou X. L. (2007). Neuroeconomics: opening the “black box” behind the economic behavior. Chin. Sci. Bull. 52, 1153–1161. doi: 10.1007/s11434-007-0193-1

[ref58] Zhang H. L. Wang L. P. Zhang X. (2021). Neuroscience in China: status quo and strategic thinking for the future. Bullet. Natl Nat. Sci. Foundation China. 35, 328–338. doi: 10.16262/j.cnki.1000-8217.2021.02.035

[ref54] Zhang S. Huang Y. F. Shi X. Z. (2021). Background and foundation analysis of construction of neuro-safety science. China Safety Sci. J. 31, 98–105. doi: 10.16265/j.cnki.issn1003-3033.2021.05.015

[ref55] Zhang S. Huang Y. F. Shi X. Z. (2022). Research on core principles of neuro-safety science. Safety Secur. 43, 36–43. doi: 10.19737/j.cnki.issn1002-3631.2022.05.006

[ref57] Zhang S. Wang S. Shi X. Z. (2023a). An EEG study of emotion mechanism in unsafe behavior decision-making. China Safety Sci J. 33, 32–40. doi: 10.16265/j.cnki.issn1003-3033.2023.01.2435

[ref59] Zhang S. Yu X. R. Shi X. Z. Zhang Y. (2023b). The influencing mechanism of incidental emotions on risk perception: evidence from event-related potential. Brain Sci. 13:486. doi: 10.3390/brainsci13030486, PMID: 36979296 PMC10046688

[ref56] Zhang X. B. Ruan M. H. Yuan T. W. Wang H. Y. Llu X. Han X.. (2020). Neuroscience and brain-inspired artificial intelligence: new progress and trends. Chin. Bull. Life Sci. 32, 993–1013. doi: 10.13376/j.cbls/2020121

[ref60] Zhao H. C. (2012). Research on miners' unsafe behavior based on physiological psychological measurement experiment. Master's thesis. Xi'an (Shaanxi): Xi'an University of Science and Technology

[ref61] Zhao Z. K. Zhang S. Shi X. Z. (2022). ERP experimental study on risk perception of safety warning signs. Safety Secur. 43, 52–59. doi: 10.19737/j.cnki.issn1002-3631.2022.09.007

[ref63] Zhou D. (2021) The research on the recruitment of professional safety managers for Subway tunnel construction based on two-sided matching between applicants and positions. Master's thesis. Changsha (Hunan): Changsha University of Science & Technology.

[ref62] Zhou P. (2016). A methodological model for cognitive neurolinguistics. J. Foreign Lang. 39, 39–47.

